# Supplementary material for: Hard- and Software Controlled Complex for Gas-Strain Monitoring of Transition Zones
Source: Sensors (Basel). 2024 Apr 18;24(8):2602. doi: 10.3390/s24082602 (PMC11053893; doi:10.3390/s24082602)
Supplement: Supplementary file 1 [file sensors-24-02602-s001.zip › sensors-2917408-supplementary.pdf]

Laser strainmeter

At Cape Schultz in the south of the Primorsky Krai of the Russian Federation a laser strainmeter of an unequal type with a measuring arm length of 52.5 m, which is oriented at an angle of 180 relative to the meridian, is installed. The central interference node is located on a concrete abutment with a height of about 3.5 m, which is fixed on hard rocks. The corner reflector is located on an abutment about 1 m high, which is firmly connected to a granite rock. All elements of the interferometer are located underground at a depth of 5 m in good hydrothermally insulated rooms. The room in which the central interference node is located is built on the principle of a thermostat with the possibility of remote conditioning of an external thermostatic room that does not contact the optics of the central interference node.

The optical scheme of the laser strainmeter is based on the scheme of the Michelson interferometer with a length of 52.5 m measuring arm, which allows measuring displacement based on the measuring arm of the laser strainmeter with an accuracy of 0.01 nm. The principle of operation of the devices consists in synchronous measurement using interference methods of changes in the phase of the laser beams passing through the measuring arms from the central interference nodes to the corner reflectors and back. In Figure 1 shows the classical optical scheme of a laser strainmeter of a non-uniform type, mainly used in measurements. Figure 2 shows a photograph of the central interference node of a 52.5-meter laser deformograph, where the distribution of optical components is shown in accordance with the diagram in Figure 1 and an underground pipeline with a diameter of 1.5 m with a vacuum-sealed stainless steel pipe in which a helium-neon laser beam propagates between the interference node and an angle reflector.

The linear operating frequency range of this strainmeter extends conditionally from 0 to 100 Hz, and at higher frequencies the amplitude-frequency response of the device varies according to the cosine law. Considering the length of the measuring arm of the laser strainmeter, it can be argued that its sensitivity is equal:

$$\Delta l/l = 0.01\ \mathrm{nm}/52.5\ \mathrm{m} \approx 0.2 \times 10^{-12}.$$

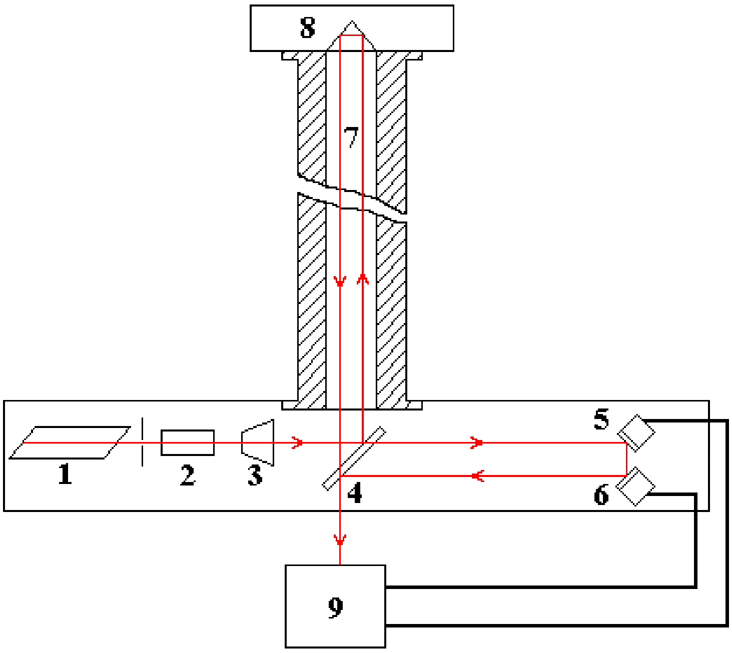

Figure S1 – Optical scheme of a laser strainmeter of a non–uniform type: 1 – laser; 2 – optical shutter; 3 – collimator; 4 - translucent plane-parallel interference plate (PI-100); 5,6 – plane-parallel alignment mirrors on piezoceramic cylinders; 7 – optical path; 8 – corner reflector; 9 – system registrations

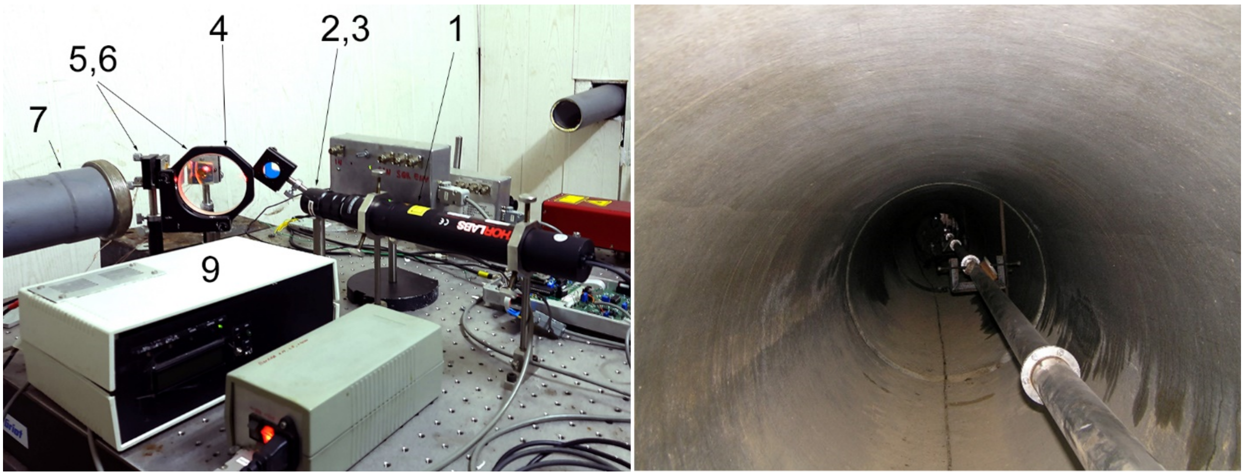

Figure S2 – The central interference node of a 52.5-meter laser strainmeter and its components in accordance with the scheme of Fig. 1. Vacuumed pipe in an underground pipeline.
